# Findings in percutaneous cholangiography in two cases of Type III cystic biliary atresia (with ultrasound correlation)

**DOI:** 10.1259/bjrcr.20150377

**Published:** 2016-05-15

**Authors:** Dimitri Parra, Annie Fecteau, Alan Daneman

**Affiliations:** ^1^ Image Guided Therapy, The Hospital for Sick Children, University of Toronto, Toronto, Canada; ^2^ Department of Diagnostic Imaging, The Hospital for Sick Children, University of Toronto, Toronto, Canada; ^3^ Division of Thoracic and General Surgery, The Hospital for Sick Children, University of Toronto, Toronto, Canada

## Abstract

Cystic biliary atresia (Type III) is uncommon and it may be difficult to differentiate this from a choledochal cyst, an entity that has a very different management and prognosis. This report shows the percutaneous cholangiographic findings in two cases of Type III biliary atresia with a non-communicating cyst, with ultrasound and clinical correlation. These findings are helpful in the diagnosis and management of patients with this condition.

## Summary

Biliary atresia (BA) is an uncommon condition with an incidence reported to be 1 in between 5000 and 19,000 live births.^1^ Its aetiology is unclear and it is classified according to the most proximal level of biliary obstruction:^1^ Type I (5%), atresia of the common bile duct; Type II (2%), atresia of the common hepatic duct; and Type III (> 90%), atresia at the porta hepatis.

Cystic biliary atresia (CBA) is a subtype that presents in 5–11% of patients with BA,^1,2^ with cysts that can be observed in an intra or/and extrahepatic location.^3^ The main differential diagnoses in a patient who presents with conjugated hyperbilirubinemia, persistent acholic stools and a cyst at the porta hepatis are CBA and infantile choledochal cyst (CC), entities with a significantly different management and prognosis.^2,3^ BA significantly benefits with a diagnosis performed before day 60–100 of life and an early portoenterostomy prevents progressive cirrhosis. A CC is a curable malformation that, if asymptomatic, can be surgically repaired in an elective fashion.^2^

The diagnostic algorithm of BA varies in different institutions. The initial imaging screening tool is usually an abdominal ultrasound. It has been reported that larger cysts, dilated intrahepatic ducts and normal gallbladder favour CC, whereas patients with smaller cysts, non-dilated intrahepatic bile ducts and abnormal gallbladder are more likely to have CBA.^2^ Sludge in the cyst has been seen more commonly in CC.^2^ Percutaneous transhepatic transcholecystic cholangiography (PTTC) has been demonstrated to safely and effectively exclude the diagnosis of BA, especially when ultrasound and hepatobiliary iminodiacetic acid scans are not conclusive.^4^ Intraoperative cholangiogram has been considered the gold standard in the diagnosis of BA and is performed prior to a Kasai portoenterostomy.^1^

In our institution, the diagnostic algorithm of BA includes an ultrasound-guided liver biopsy and a PTTC when technically feasible to perform during the same anaesthesia. Informed consent is obtained prior to the procedures. The interventions are performed under general anaesthesia, sterile technique and using prophylactic antibiotic. The PTTC is usually performed prior to the biopsy. The gallbladder is identified and accessed under ultrasound guidance, using a 22-, 25- or 27-gauge needle (according to the operator’s preference). Contrast is injected and different fluoroscopy projections are obtained. When the study is completed, the residual contrast is aspirated from the gallbladder. This study is followed by the ultrasound-guided biopsy that is performed using a coaxial technique, utilizing a 17-gauge coaxial needle and an 18-gauge biopsy device, and embolizing the tract with one or two pledgets of the Hunter biopsy sealing device (Vascular Solutions Inc., Minneapolis, MN) or Gelfoam slurry (Pharmacia & Upjohn Company, Kalamazoo, MI). The biopsy is performed using a second puncture site, usually in the left liver lobe.

Risks of the procedures include bleeding, infection (cholangitis), injury to the liver, bile leak, arteriovenous fistula formation, and they are explained to the caregiver at the time of obtaining the informed consent.

We present two consecutive cases of CBA, in which the PTTC and sonographic findings were very useful in pre-surgical planning, showing findings consistent with Type III BA with a non-communicating extrahepatic cyst.

## Findings

### Case 1

**Figure 1.**
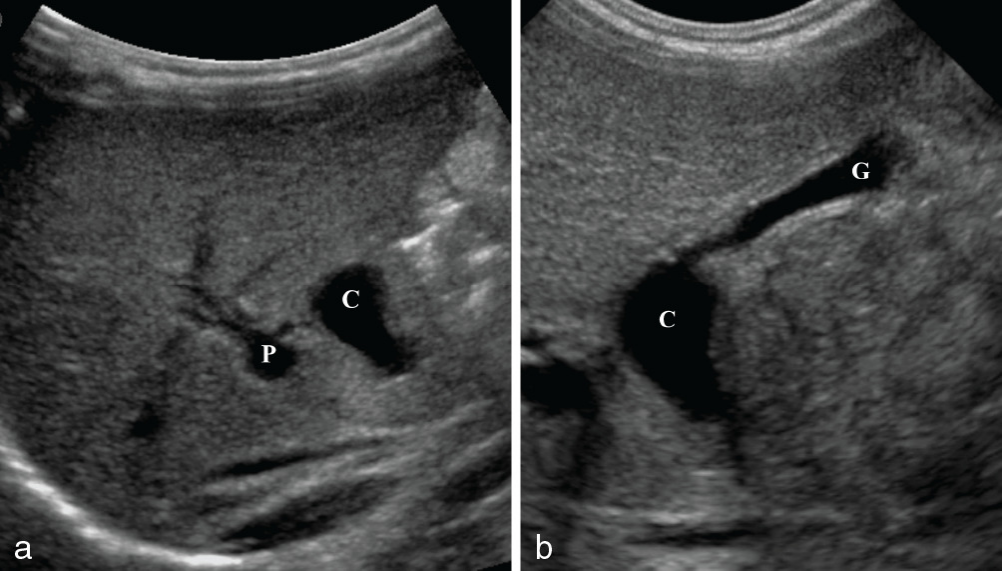
Ultrasound of the abdomen of case 1 showing (a) an anechoic extrahepatic cystic structure (C) adjacent to the main portal vein (P). (b) This cyst (C) was connected to a small and elongated gallbladder (G).

**Figure 2.**
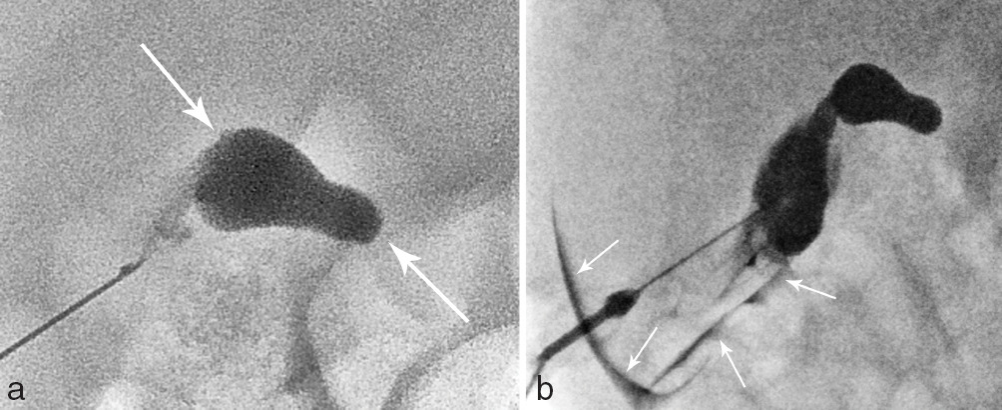
Percutaneous transhepatic transcholecystic cholangiography findings in case 1. (a) Prompt opacification of the known extrahepatic cyst (arrows), which did not show communication with the bile ducts or the duodenum. (b) After sustained injection of contrast, there was a contained retroperitoneal perforation of the cyst/gallbladder (arrows) and no opacification of the bile ducts or the bowel.

A 25-day-old term baby female presented with neonatal jaundice and hyperbilirubinemia. A biliary cyst and a contracted gallbladder were diagnosed in a post-natal ultrasound from another institution. She was transferred to our hospital at day 19 of life. A repeat ultrasound showed a small gallbladder (length 1.7 cm; diameter 0.3 cm) connected to an extrahepatic cystic structure, which measured 1.4 × 0.6 cm (Figure 1). The content of the cyst was anechoic and there was no dilatation of intrahepatic bile ducts. A hepatobiliary iminodiacetic acid scan on day 26 of life showed no biliary drainage up to 28 h. A PTTC on day 35 of life showed there was prompt opacification of the known extrahepatic cyst, followed by visualization of a small gallbladder. There was no passage of contrast into bile ducts or the duodenum. Aiming to demonstrate that there was no communication of this cyst with the biliary system as well as to mechanically remove any source of obstruction, the contrast injection was sustained until there was an extraperitoneal perforation of the cyst (Figure 2) (radiation dose: 1.1 mGy). The rationale to sustain the injection was to demonstrate a connection of the cyst with the biliary tree and our experience that there may be a benefit of mechanical lavage of the biliary tract in cases of obstruction by secretions or sludge, which has been reported by our institution in neonates with parenteral nutrition-related cholestasis.^5^ An uncomplicated ultrasound-guided biopsy was performed. Intravenous antibiotics were started owing to perforation of the cyst and the patient recovered well from the procedures. The biopsy result was consistent with extrahepatic biliary obstruction.

At the time of surgery, a cystic structure, distinct from the gallbladder, was encountered, which was located in the common bile duct. Proximally, the cyst was in continuity with a cord-like hepatic duct. A standard Kasaï procedure was performed with a hepaticojejunostomy. Subsequently, the patient’s jaundice resolved.

### Case 2

**Figure 3.**
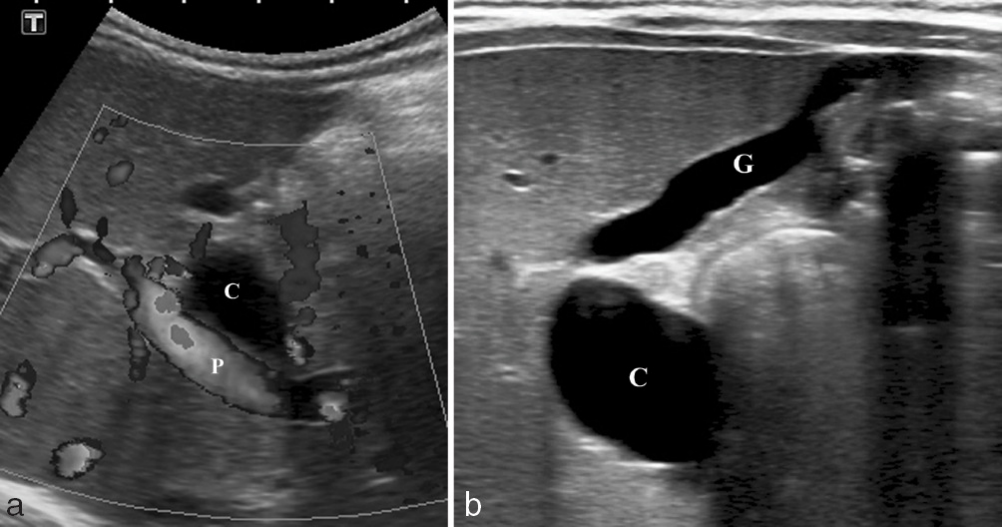
Ultrasound of the abdomen of case 2 showing (a) a large anechoic cyst (C), without colour Doppler signal, adjacent to the main portal vein (P). (b) The cyst (C) was connected to a gallbladder (G) with an abnormal appearance (small and elongated).

**Figure 4.**
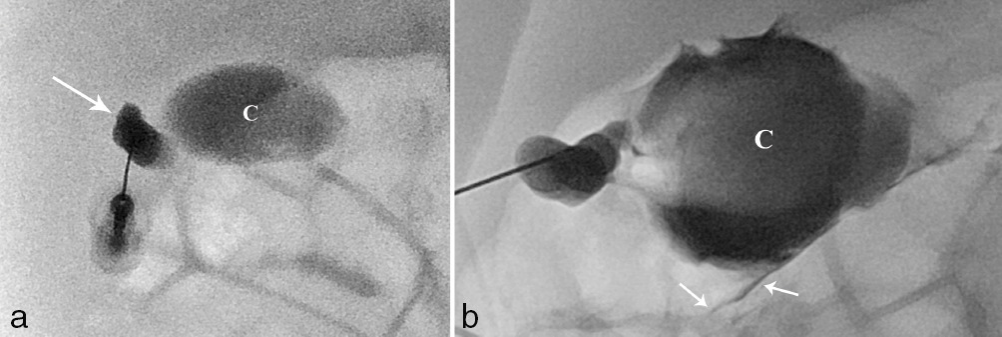
Percutaneous transhepatic transcholecystic cholangiography findings in case 2. (a) Opacification of a small gallbladder (arrow) connected to a large cyst (C). (b) Sustained contrast injection distended the cyst (C). No communication with the bile ducts or the bowel was demonstrated. The injection was stopped as soon as a contained retroperitoneal perforation was noted (arrows).

A 51-day-old term baby male presented with neonatal jaundice, hyperbilirubinemia and abnormal liver function tests. Ultrasound showed a cystic structure anterior to the portal vein measuring 2.3 × 1.6 × 1.8 cm. The gallbladder had an unusual, small, irregular appearance measuring 3.9 cm in length and 0.6 cm in diameter (Figure 3). There was no sludge in the cyst and no intrahepatic bile duct dilatation. A PTTC on day 56 of life showed opacification of a small gallbladder connected to a prominent cystic structure (Figure 4). No passage of contrast into the bile ducts or the duodenum was observed. To definitively exclude a connection of the cyst, the contrast injection was maintained until there was an extraperitoneal perforation of the cyst (Figure 2) (radiation dose: 2.3 mGy). An uneventful ultrasound-guided biopsy was then performed. Intravenous antibiotics were started and the patient recovered well from the procedure. The biopsy result was consistent with large duct obstruction, with early signs of liver fibrosis.

Dissection of the porta hepatis at the time of surgery revealed cystic dilatation of the common bile duct without communication to the duodenum and a fibrotic hepatic duct proximally. This was resected at the level of the portal plate and a Kasai hepaticojejunostomy was performed. The child’s jaundice resolved postoperatively.

## Discussion

In a neonate with cholestasis and a portal cyst, the differential diagnosis includes CBA and CC. The management and outcome of these two conditions is dramatically different: BA requires early portoenterostomy to prevent progressive cirrhosis; CC can be surgically repaired in an elective fashion.

The cases presented illustrate CBA, an infrequent presentation of BA. The initial ultrasound showed an extrahepatic cyst associated with an unusual appearance of the gallbladder in terms of size and morphology. In both the cases, there was no sludge in the cyst and no intrahepatic biliary duct dilatation, findings described as favouring CBA.

The PTTC showed a small gallbladder connected to a “non-communicating” cyst as described in CBA. The biopsy results supported the findings of BA. The diagnosis was confirmed at surgery and both patients had a successful Kasai portoenterostomy. In our experience, the diagnostic work-up and management of CBA is the same as the one for isolated BA.

In conclusion, this report illustrates the typical findings in PTTC and ultrasound of CBA, an infrequent condition that needs to be considered in the differential diagnosis of a neonate with cholestasis and an extrahepatic cyst. Immediate recognition and effective communication of the ultrasound findings between the radiology, and the medical and surgical teams as well as judicious use of PTTC is fundamental for the diagnosis and management of this condition.

## Learning points

1. The cystic type is an uncommon presentation of BA; however, it is important for radiologists to be aware of it.
2. PTTC can be very useful in the diagnostic algorithm of BA. CBA may have distinctive features in this imaging modality.
3. CBA and CC have significantly different management; therefore, it is very important to differentiate them from one another.

## Consent

This manuscript includes two cases and informed consent was obtained from the legal guardians of both patients for this publication.
